# Nelarabine Associated Myotoxicity and Rhabdomyolysis

**DOI:** 10.1155/2015/825670

**Published:** 2015-03-18

**Authors:** Mahnur Haider, Syed Ahsan Rizvi, Pashtoon Murtaza Kasi

**Affiliations:** Division of Internal Medicine, Mayo Clinic, 200 1st Street SW, Rochester, MN 55905, USA

## Abstract

Nelarabine (ara-G; Arranon; compound 506U78) is an antineoplastic purine analog used for the treatment of refractory or relapsed T-cell acute lymphoblastic leukemia (T-ALL) and T-cell lymphoblastic lymphoma (T-LBL). The drug was granted accelerated approval in October 2005 by the US Food and Drug Administration (FDA) given the efficacy (induction of complete responses) noted in 2 single-arm trials (one in pediatric setting and one in adult patient population). The main spectra of toxicities that have been reported in these clinical trials and subsequent studies are hematological and neurological. Nelarabine induced rhabdomyolysis and increased creatinine phosphokinase (CK; CPK) levels apparently have been reported and this side effect has been added as an adverse reaction in the product monograph from the drug company during postmarketing surveillance. However, the true extent and incidence of the myotoxicity from the drug are unclear. In this paper we report a grade IV CK elevation and rhabdomyolysis in a patient with T-ALL treated with nelarabine. Given the reported finding, we examined the literature further for myotoxicity, increased CK, and/or rhabdomyolysis associated with the use of the nelarabine and report our findings.

## 1. Introduction

Nelarabine (ara-G; Arranon; compound GW506U78) is an antineoplastic purine analog used for the treatment of refractory or relapsed T-cell acute lymphoblastic leukemia (T-ALL) and T-cell lymphoblastic lymphoma (T-LBL) [1]. The drug was granted accelerated approval in October 2005 by the US Food and Drug Administration given the efficacy (induction of complete responses) noted in 2 single-arm trials (one in pediatric setting and one in adult patient population) [1].

Nelarabine is a prodrug that is converted to 9-*β*- D arabinofuranosylguanine (ara-G) after demethylation by adenosine deaminase and is more cytotoxic to T-cells than B cells [2]. The main spectra of toxicities that have been reported in the original clinical trials and subsequent studies are hematological and neurological. With increasing use, other toxicities and side effects are being recognized.

In this paper we report a grade IV creatinine phosphokinase (CK; CPK) increase and rhabdomyolysis in a patient treated with nelarabine. Given the reported finding, we examined the literature further for myotoxicity, increased CK, and/or rhabdomyolysis associated with the use of the nelarabine and report our findings.

## 2. Case Presentation

A 33-year-old Caucasian male was diagnosed with T-cell acute lymphoblastic lymphoma and leukemia in August 2013, after a mediastinal mass was detected on a chest X-ray to evaluate the cause of a persistent cough. A computerized tomography (CT) guided biopsy of the mass confirmed the diagnosis of T lymphoblastic leukemia/lymphoma with abnormal blastic lymphoid population positive for CD1a, CD2, CD3, CD4, C5, C7, CD8, CD 10, and CD45, TdT, and PCR beta, which supported the diagnosis. Bone marrow biopsy showed 10% involvement of a normocellular to hypocellular marrow. A positron emission tomography-computed tomography (PET/CT) scan was negative for any other sites of disease apart from the mass as noted above.

Chemotherapy in the form of hyper-CVAD (cyclophosphamide, vincristine, doxorubicin (also known by its trade name, adriamycin), and dexamethasone alternating with methotrexate and cytarabine) was initiated in September 2013 and after completion of the 8 cycles of therapy, restaging bone marrow biopsy unfortunately showed recurrent T-ALL involving 70% of the bone marrow cellularity.

Reinduction therapy was initiated in June 2014 and he received his first dose of nelarabine on day 1 followed by a dose on day 3 and day 5 as per protocol. First cycle was complicated by neutropenic fever requiring intravenous (IV) antibiotics. Patient then went on to receive cycle 2 as per protocol. However, 2 days later after completion of chemotherapy, when he returned for ongoing follow-up, he was noted to have bilateral calf pain which rapidly became severe and incapacitating.

**Figure 1:**
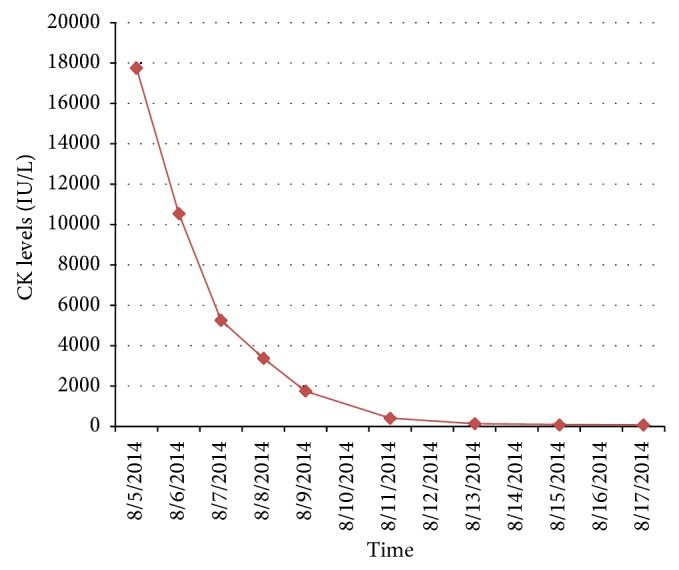
Trend in the elevation in CK levels noted after second cycle of chemotherapy with nelarabine in our patient (note: cycle 1 was initiated on 6/27/2014 and cycle 2 on 07/29/2014; last dose was given 08/02/2014, no baseline CK levels to compare to).

On physical exam he had marked tenderness of both calves. His laboratory findings showed a serum lactate dehydrogenase (LDH) elevated at 717 U/L; aspartate transaminase (AST) and alanine transaminase (ALT) that was previously normal (23 U/L and 55 U/L, resp.) were increased to 403 U/L and 94 U/L, respectively. Urine analysis was positive for trace hemoglobin. Serum potassium, phosphorous, calcium, creatinine, BUN, and anion gap were within the normal range and Doppler ultrasound of the legs was negative for deep vein thrombosis. CK levels are checked on admission and were noted to be markedly elevated to 17,757 IU/L (Figure 1). Given the diagnosis of rhabdomyolysis and severe symptoms, he was managed with aggressive IV hydration and analgesic support. There were no sequelae secondary to the rhabdomyolysis and patient was discharged after a 5-day hospital stay.

Patient then proceeded to a matched unrelated-donor, allogeneic stem cell transplant. His leukemia unfortunately relapsed on day +63 after transplant. Bone marrow biopsy showed 72% blasts. He was started on dose reduced clofarabine and cytarabine due to acute kidney injury. His course was complicated by respiratory distress and persistent fevers despite broad-spectrum antibiotics, which were attributed to CMV pneumonitis. His disease continued to progress and performance status declined. Goals of care were readdressed and patient was transitioned to hospice and died 4 months after transplant.

## 3. Discussion

“Rhabdomyolysis is a well-known clinical syndrome of muscle injury associated with myoglobinuria, electrolyte abnormalities, and often acute kidney injury (AKI) [3].” Typical symptoms include pain, weakness, tenderness, and/or swelling of the muscles injured. It can also present with ambiguous symptoms of fatigue, nausea, vomiting, and fever. As noted by Zimmerman and colleagues, apart from the known causes like trauma, rhabdomyolysis in the patient population is now more due to prescription and/or over the counter medications. Drugs that are well known for myotoxicity and rhabdomyolysis include HMG-CoA reductase inhibitors, antiepileptics, and some antimicrobials (like trimethoprim-sulfamethoxazole (Bactrim) and quinolones). Some of the newer tyrosine kinase inhibitors (TKIs), for example, Sunitinib and Erlotinib used in the treatment of some cancers, have also been associated with increasing CK levels and/or rhabdomyolysis.

With respect to nelarabine, the data and reviews on the drug mainly focus on the neurological and hematological side effects. The neurotoxicity from nelarabine can be severe and can manifest itself in the form of both peripheral and central neuropathy [4–6]. This could range from dysautonomia, paresthesias, peripheral sensory neuropathy, ataxia, somnolence, and/or seizures [7–9]. This tends to be cumulative and concomitant use with other intrathecal chemotherapies should be avoided [4, 10, 11].

Nelarabine induced rhabdomyolysis and increased CK levels apparently have been reported and this side effect has been added as a postmarketing adverse reaction in the product monograph from the drug company [12]. However, the true extent and incidence of the myotoxicity from the drug is unclear.

We extensively reviewed the available published results of the phase I/II studies and also searched the literature for any reported case reports pertaining to nelarabine associated CK elevation and/or rhabdomyolysis. We found that in one of the main Phase II studies published in the Journal of Clinical Oncology in 2011, the authors noted, “surprisingly, 1 patient developed pronounced increases of creatine kinase (grade IV), which later decreased without sequelae [13].” Additionally, in earlier studies on nelarabine, musculoskeletal symptoms have been reported without an “identifiable” cause [14, 15]. In the Cancer and Leukemia Group B study (CALGB) 19801 study, note was made of 4 (11%) patients developing muscle weakness and 1 (3%) patient developing grade 3 myalgia as well. No mentioning of rhabdomyolysis or high CK levels was made [16].

This is an important finding. Looking closely at the adverse events reported in the clinical trials on nelarabine, there are definitely a proportion of patients who have grade 3 or more musculoskeletal related side effects. These range from muscle weakness to myalgias to isolated cases of rhabdomyolysis as noted. Although it is difficult to extrapolate, but these may well secondary be due to undiagnosed myotoxicity from the drug in a subset of patients. Moreover, there are also within the same trials reports of aspartate aminotransferase (AST) grade 3/4 increases in a subset of patients [17, 18]. It is possible that AST rise may be coming from muscle breakdown; however, without having simultaneous CK levels checked for these patients, it is difficult to make this assumption.

Our patient was diagnosed with rhabdomyolysis after getting his second cycle of nelarabine. This was temporally associated to nelarabine given the lack of any other factors (such as immobility or trauma) and/or lack of exposure to any other new medications that have been associated with rhabdomyolysis. He, given the chemotherapy and immunosuppression, was on multiple medications, which included cefepime, docusate, enoxaparin, granisetron, lorazepam, metoprolol, prochlorperazine, Bactrim, valacyclovir, and voriconazole. Out of these medications Bactrim is the only medication rarely associated with rhabdomyolysis. The diagnosis of nelarabine induced rhabdomyolysis was made given the chronological and temporal sequence of events. As to why it happened after cycle 2 and not cycle 1 is unclear to us. Additionally, CK levels are not routinely checked unless there are symptoms, so it is possible that there might have been some myotoxicity even after cycle 1. Symptoms in our patient's case developed after the initiation of the drug and the patients' condition improved on withholding the drug. With respect to Bactrim, the patient had been taking this for prophylaxis for an extended period of time and in the past had taken this medication without the development of any side effects. During his hospital stay he continued to receive Bactrim and was discharged with it making it an improbable cause of rhabdomyolysis.

Therefore, we consider our patient's rhabdomyolysis to be induced by nelarabine because of the temporal sequence of events, as nelarabine was the only medication that had recently been introduced and the patient's condition improved without the discontinuation of other medications. According to the Naranjo adverse drug reaction probability scale, nelarabine would score ~7, which would put in the “*probable*” cause of the adverse reaction.

A diagnosis of rhabdomyolysis can be made with a creatinine kinase level greater than 500 IU/L or 5 to 10 times the upper limit of normal, with or without clinical symptoms. Creatinine kinase serum level is one of the specific tests and can be correlated with the amount of muscle injury, however, as in our patient as well, the exact level is not necessarily indicative of the development of complications. Checking aldolase levels in these patients may also be helpful. Our patient fortunately did not develop any rhabdomyolysis related complications, even though creatinine kinase level was markedly elevated and was given early aggressive supportive management.

Postmarketing surveillance and our review showed that nelarabine is associated with cases of elevations of CK and rhabdomyolysis. Discontinuation of nelarabine therapy should be considered if rhabdomyolysis and elevated CK are suspected to be associated with the use of the drug alongside providing aggressive supportive management. We do not believe that there is a particular CK level that would warrant discontinuation of therapy; however, it would be important to consider myotoxicity/rhabdomyolysis as a potential etiology early in the course of any patient presenting with myalgias, muscle weakness, out of proportion fatigue, and/or unexplained acute kidney injury after having received nelarabine. Checking of baseline CK levels and CK levels prior to administration of subsequent cycles may help identify the potential subset of patients prone to developing the myotoxicity and give a true estimate of the burden of disease.
